# Intensity is in the eye of the beholder: a qualitative photovoice study of perceptions of physical activity intensity for people with chronic obstructive pulmonary disease

**DOI:** 10.1186/s12890-026-04520-8

**Published:** 2026-07-21

**Authors:** Phoebe H.I. Lloyd-Evans, Dominic Malcolm, Sally J. Singh, Alex V. Rowlands, Emma Chaplin, Sarah Ward, Mark W. Orme

**Affiliations:** 1 Department of Respiratory Sciences, University of Leicester, Leicester, UK; 2 Centre for Exercise and Rehabilitation Science (CERS), NIHR Leicester Biomedical Research Centre-Respiratory, University Hospitals of Leicester NHS Trust, Leicester, UK; 3 Assessment of Movement Behaviour Group (AMBer), Leicester Lifestyle and Health Research Group, Diabetes Research Centre, University of Leicester, Leicester, UK; 4 School of Sport, Exercise and Health Sciences, Loughborough University, Loughborough, UK; 5 NIHR Leicester Biomedical Research Centre, Leicester, UK; 6 Alliance for Research in Exercise, Nutrition and Activity (ARENA), UniSA Allied Health and Human Performance, University of South Australia, Adelaide, Australia

**Keywords:** Physical activity, Intensity, Duration, Chronic Obstructive Pulmonary Disease

## Abstract

**Background:**

Physical activity (PA)-related health benefits are greatest for activity undertaken at higher intensities. However, individuals living with Chronic Obstructive Pulmonary Disease (COPD) often find engaging in higher-intensity PA challenging. Due to the lack of qualitative research on how those with COPD perceive PA intensity, this photovoice study explores how PA intensity is experienced in day-to-day living.

**Methods:**

Semi-structured interviews, enhanced by photovoice, were conducted with individuals with COPD and other comorbidities to explore their perceptions of PA intensity in daily life. Participants captured images over 7 days to illustrate how their conditions impact their PA. The participant-generated images were used in the interviews. Reflexive inductive thematic analysis was used to generate themes.

**Results:**

Thirteen interviews were conducted (54% female, aged 64 ± 6 years), generating two main themes. Theme 1 focused on how individuals employ strategies to manage the intensity of their PA: breaking down PA, pacing themselves or trying to do the activity all in one go. Theme 2 highlighted the reciprocal relationship between participants and their family/friends regarding PA intensity. Family/friends often ensure individuals do not “overdo it” while participants may influence the intensity of their family/friends activities, sometimes requiring family/friends to slow down or stop.

**Conclusion:**

This study provides insights into the social complexities and strategies used to manage PA intensity in daily life, which can inform behavioural interventions to increase participation in higher-intensity PA among people with COPD.

**Trial registration:**

The study was retrospectively registered on Clinical Trials.gov (Identifier: NCT06330623) on the 26th of March 2024.

**Supplementary Information:**

The online version contains supplementary material available at https://doi.org/10.1186/s12890-026-04520-8.

## Background

Regular physical activity (PA) has been shown to provide significant health benefits, particularly in preventing and managing chronic conditions [1–3]. A recent narrative review by Ekelund et al. [4] reported greater health benefits associated with higher intensity PA, even if the volume was similar, i.e., shorter duration vigorous PA was more beneficial than longer duration moderate PA. The intensity of PA refers to the energy demand of an activity [5], expressed in either absolute (i.e., the energy expenditure required to perform a certain activity) or relative terms (i.e., the ease or difficulty of the activity based on an individual’s perception) [6]. However, the same absolute intensity of PA could be perceived as light by one individual and as moderate-to-vigorous intensity by another individual, depending on personal characteristics such as age, physical capacity, health status, social and biographical factors [7, 8].

People living with COPD often experience a variety of debilitating symptoms, such as pain, breathlessness and fatigue [9, 10], which can exacerbate perceptions and experiences of absolute and perceived PA intensity, interacting with the effects of age-related change [11], thus influencing the difficulty of daily activities. People living with chronic respiratory diseases, such as COPD, epitomise many of the challenges of living with chronic disease, including having a high symptom burden, being less physically active and exhibiting a reduced physical capacity [12, 13] compared to healthy adults [14] and even those living with many other long-term chronic conditions [15].

The way a person perceives difficulties associated with the intensity of their PA, while in part determined by a person’s maximal capacity (calculated as relative intensity), is a subjective or qualitative concept. Consequently, the notion of relative intensity is widespread in exercise studies. More recently, it has been increasingly recognised in quantitative PA studies using accelerometry and attempting to account for individuals’ physical capabilities in the measurement of intensity [7, 11–13]. Despite this, there is currently limited qualitative exploration of how a person perceives their PA intensity, particularly in those with COPD. Photovoice can be used to foreground the subjective nature of PA intensity, providing real-world context by combining photographs taken by participants with words [16]. Participants are empowered through photography by choosing what images to capture and discuss. It allows participants to highlight the factors which they themselves identify as most significant and to express themselves in ways that may be difficult to articulate with words alone [16].

Previous research using photovoice has explored the experiences of individuals living with chronic respiratory diseases (CRDs) [16–20], highlighting environmental factors and other barriers to lifestyle behaviours, such as PA [21–23]. However, these studies tend to focus on the broad experiential themes rather than the specific ways individuals with CRDs interpret and regulate the intensity of their PA. Recognising this gap reinforces the importance for investigating perceived PA intensity. By employing photovoice, this study aimed to build upon existing research on PA among individuals with COPD. Specifically, this qualitative photovoice study aimed to explore the perceptions of lived experiences of PA intensity in a comorbid population living with COPD.

## Methods

### Study design

This is a cross-sectional qualitative study, founded on grounded theory, using semi-structured interviews and photovoice. This study reported in accordance with the Consolidated Criteria for Reporting Qualitative Research (COREQ) checklist [24] (Supplementary material, Table S1).

### Ethical approval

Ethical approval was provided by the Health and Social Care Research Ethics Committee. (REC: 23/NI/0130). All participants provided written informed consent.

### Recruitment and participants

Participants were recruited from the pulmonary rehabilitation (PR) service, before taking part in PR and from an existing research database of participants from previous studies at the University Hospitals of Leicester NHS Trust. Recruitment took place between January 2024 and May 2024. For participants recruited from the PR service, the PR team identified potential participants during routine telephone assessments and then referred them to the research team. Following this, a doctoral researcher [PLE] explained the study to possible participants. If they wanted to participate, a baseline visit was organised. A purposive sampling technique was used for this study. Inclusion criteria involved participants being aged ≥ 40 years and having a confirmed diagnosis of COPD, using spirometry guided by the Global Initiative for Chronic Obstructive Lung Disease (GOLD) guidelines [9].

### Data collection – photovoice approach

#### Step 1: Baseline visit

Participants attended a baseline visit with PLE, where written informed consent was obtained. The purpose of this visit was to establish rapport with participants and provide them with instructions on photo taking. Demographic data were collected from the participants’ medical records, including age, gender, ethnicity, and comorbidities.

#### Step 2: Photo taking

Before taking part in interviews, participants were provided with a digital camera and asked to take photographs, for a 7-day period, that represent how their COPD impacts the intensity of their PA and daily activities. Participants were told that there was no limit on the number of photos they could take. Participants could still participate in the interviews even if they had not captured any images. PLE accessed the photos prior to the interview, while the participants were completing study-related questionnaires.

#### Step 3: Interview

Semi-structured interviews were conducted by a female doctoral researcher trained in qualitative interview techniques [PLE (MSc)]. PLE piloted the interview schedule before conducting the study. Interviews were conducted in person in a private room in the hospital or over a video call, according to participant preference. Interviews were audio-recorded with a digital voice recorder, transcribed verbatim and anonymised. A semi-structured interview guide was used (as shown in supplementary material, Table S2). For each photograph taken by participants, they were asked questions about what the image represents to them in the context of their COPD and how it impacts the intensity of their PA. Field notes were made following each interview in order to capture immediate reflections and were used as part of the reflexive thematic analysis. Before each interview, participants completed the Medical Research Council (MRC) Dyspnoea Scale [25], which assesses functional disability due to dyspnoea, and the COPD Assessment Test (CAT) [26], which quantifies the impact of COPD symptoms on a patient’s overall health.

### Analysis

Reflexive thematic analysis of the transcripts was conducted in line with Braun and Clarke [27]. Reflexive thematic analysis was used to investigate the complex and subjective experiences of PA intensity in individuals with COPD. This approach was selected to enable the study to go beyond mere description of PA, allowing interpretation of how individuals with COPD perceive the intensity of their daily activities. The analysis involved reading each transcript closely, identifying patterns, labelling codes to data, and generating themes and sub-themes [28]. The analysis utilised a bottom-up approach to inductively derive themes from the interviews. PLE coded and analysed the transcripts to identify and classify categories from the data related to the research question. Photographs were used as discussion stimuli rather than as data in themselves [29], enabling participants to articulate the phenomena and meaning of COPD and PA intensity in their own voices. Transcripts were not returned to participants for comments. Feedback from two of the other authors [DM (PhD), MO (PhD)] on coding and analysing segments of data and themes was given. DM has extensive experience in conducting and supervising qualitative research with physically active populations.

Theme development was collaboratively reviewed over four monthly meetings, three one-to-one meetings between PLE and DM, and three one-to-one meetings between PLE and MO. The aim of these meetings was to challenge assumptions, conceptualise findings, and ensure that the themes accurately represented the dataset rather than reflecting only individual interpretations. We considered which interpretations align best with the wider findings, as qualitative findings are interdependent rather than just ‘free-floating’ data extracts. Disagreements in coding were addressed through discussions among the authors until a consensus was reached. Analysis was conducted after every three interviews.

We confirm information power was achieved following 13 interviews, supported by the application of established theory during analysis and high quality of dialogue during data collection, which ensured rich, meaningful insights from a relatively small sample [30]. The last 3 interviews were used to critically reflect on the themes and sub-themes generated. Our sample size is comparable to other studies using photovoice to explore PA in CRD populations [21, 22].

### Reflexivity

It was acknowledged that backgrounds, biases, and experiences could impact this study [31].

To address potential biases, we implemented several approaches in this study. Firstly, PLE documented field notes subsequent to each interview. This practice enabled her to reflect critically on her insights and to assess any potential biases. Moreover, PLE applied open and non-leading questioning while maintaining clear boundaries between personal experience and analytic decisions. Additionally, coding decisions were systematically reviewed during four monthly meetings, and a thorough audit trail was maintained to document code refinements. This helped to ‘sense check’ the interpretation of data extracts, providing an additional layer to this reflexive approach, and helped maintain the integrity and rigour of the study.

At the time of the interviews, PLE was completing her PhD and held an MSc in Exercise as Medicine and a BSc in Sport and Exercise. Although younger than most participants (26 years old), PLE’s White British background aligned with the majority of participants in the study. PLE received training in qualitative methods (interviewing and thematic analysis training), establishing a strong foundation for the analytic approach adopted in this study. PLE had prior engagement with patients with COPD by attending public patient involvement meetings, which enhanced her communication skills and deepened her understanding of their experiences of living with COPD and PA.

## Results

### Participants

Fourteen participants were recruited for this study, with one subsequently dropping out due to a lack of time to participate. Thirteen interviews were completed (*n* = 11 face-to-face and *n* = 2 video calls; 54% female, aged 64 ± 6 years, 69% MRC 3: “I walk slower than people of the same age on the level because of breathlessness, or I have to stop for breath when walking at my own pace on the level”, 92% CAT ≥ 10) (Table 1). Twelve participants (92%) provided photos (*n* = 3–89). Interviews lasted 30–75 min. All participants had comorbidities, of which 85% had two or more comorbidities (85% musculoskeletal, 62% gastrointestinal, 46% metabolic, 39% vascular, 39% psychiatric, 15% cardiac and 15% cancer).

**Table 1:** Descriptive characteristics of the sample population

Identifier	Age (yrs.)	Sex	Ethnicity	MRC	CAT	Severity of Airflow Obstruction	BMI
P1	70	Male	White British	2	12	Moderate	34.5
P2	61	Female	White British	3	31	Moderate	41.8
P3	56	Female	White British	5	23	Moderate	23.6
P4	58	Female	White British	3	32	Mild	41.9
P5	66	Male	White British	3	24	Moderate	NA
P6	56	Male	British Caribbean	3	28	Severe	29.7
P7	66	Female	White British	3	27	Severe	39
P8	64	Male	White British	3	26	Moderate	31.9
P9	61	Male	White British	2	13	Mild	29.3
P10	70	Female	White British	2	9	Mild	26.2
P11	62	Female	White British	3	27	NA	25.3
P12	75	Male	White British	3	22	Moderate	33.9
P13	68	Female	White British	3	19	Moderate	NA

*MRC* Medical Research Council Dyspnoea Scale, scale ranges from 1 to 5 to establish the clinical grades of breathlessness, MRC 2: getting short of breath when hurrying on the level or walking up a slight hill. MRC 3: walking slower than people of the same age on the level because of breathlessness, or having to stop for breath when walking at my own pace on the level. MRC 5: too breathless to leave the house or breathless when dressing. *CAT* COPD Assessment Test, scores range from 0 to 40, with higher scores indicating more symptoms. Domains: cough, phlegm, chest tightness, breathlessness, activities, confidence, sleep and energy. Severity of Airflow Obstruction: GOLD COPD severity classifies airflow obstruction into four stages (1–4) based on spirometry: GOLD 1 (Mild): *FEV*1 ≥ 80% predicted; GOLD 2 (Moderate): 50% ≤ *FEV*1 < 80% predicted; GOLD 3 (Severe): 30% ≤ *FEV*1 < 50% predicted and GOLD 4 (Very Severe): *FEV*1 < 30% predicted. *BMI* Body mass index, expressed as kg/m^2^. *NA* Data not available/missing

Two main themes were generated: (1) Approaches to manage the intensity of their PA and (2) Relationships between PA and family/friends. Participant quotes are interweaved throughout the results section for illustrative purposes, with participants indicated as P1-13. We also include photos to support and provide context for participants’ quotes.

### Theme 1: Approaches to manage the intensity of their physical activity (PA)

Theme 1 describes the approaches participants used to manage the intensity of their PA. Three sub-themes were generated: (1.1) “break it down”, (1.2) “pacing myself”, and (1.3) “achieve it all in one go”.

#### Subtheme 1.1: ‘Break it down.’

**Fig. 1:**
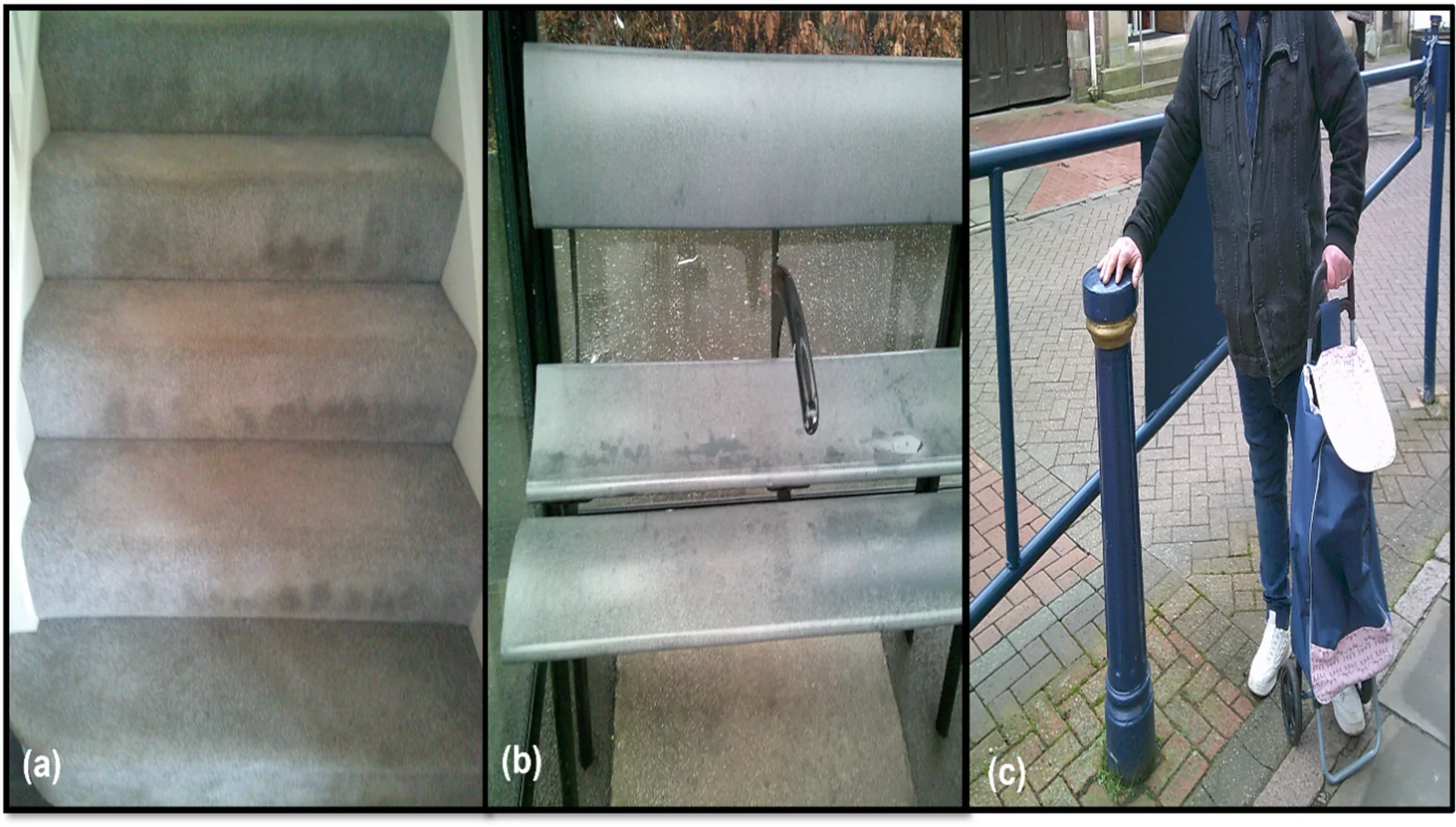
Examples of breaking up activities with rest, such as (**a**) resting after so many steps on the stairs, (**b**) sitting on a bench and (**c**) resting on a pillar

Participants provided photographs to illustrate and emphasise the importance of taking breaks to prevent the intensity of PA going too high. One participant explained that when walking up the stairs, they ‘count so many [steps], stop and then do so many more’ (P11) (Fig. 1a). A second participant described using a local bus stop bench to ‘sit down for a minute’ to catch their breath after going up a hill on their triweekly walk (P2) (Fig. 1b). Another resting aid that a participant identified was the use of a pillar to lean against, rest and then continue on their walk (P5) (Fig. 1c). It was noted that participants use resting spots as part of their daily routine. One participant stated, ‘Quite often, I’ll stop halfway through [walking] and have…a coffee’ (P1). The participant continued to state, ‘I don’t throw myself’ into PA (P1).

The rationale for breaking down PA varied among participants, with some individuals feeling that becoming breathless and needing to break up activity with rest is ‘a natural process’ and their breath ‘always comes back’ (P9). In contrast, others felt ‘frustrated’ (P11) having to sit down and rest during activity as they would compare their current ability (primarily limited by their symptoms) to their perceived ability before being limited by their symptoms, when ‘they wouldn’t have done that’ and would have instead ‘just kept going’ (P11). Several participants felt ‘a bit embarrassed’ (P4) as they would compare their current ability to the perceived ability of someone of a similar age without COPD, stating that they did not want to rest because they felt they ‘shouldn’t have to’ (P4) at their age.

#### Subtheme 1.2: ‘Pacing myself’

**Fig. 2:**
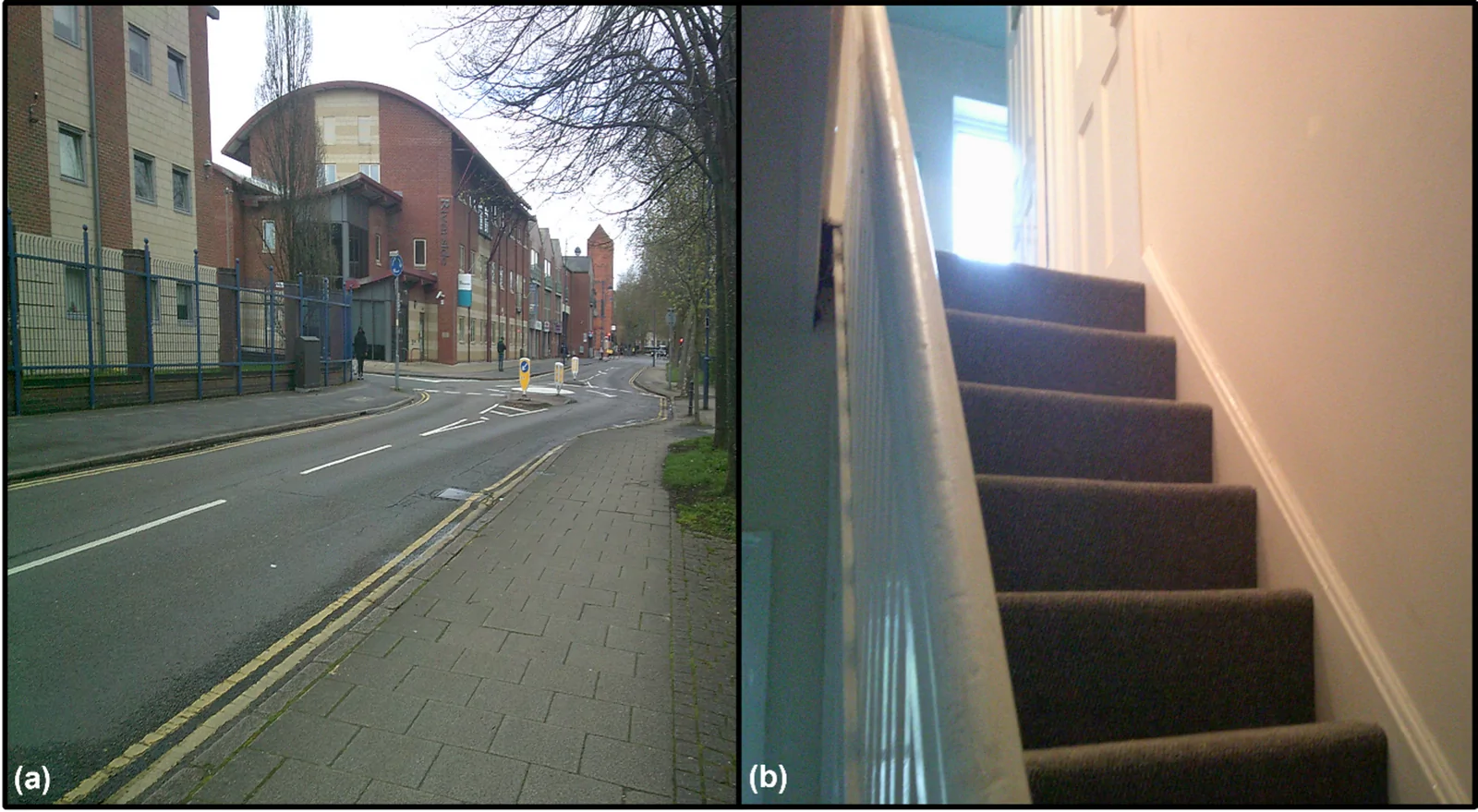
Examples of using pacing during activities, such as (**a**) pacing when walking outside between the doctors’ surgery and the pharmacy practice and (**b**) pacing when walking up the stairs

Several participants highlighted the importance of pacing themselves when doing more intense PA, downgrading the intensity of an activity and avoiding the need to stop or rest. One participant stated, ‘I’ll get so far and slow down a bit and try…. not to be too out of breath and try and get to where I’m going’ (P7), such as walking from the local surgery to the pharmacy (Fig. 2a). A second participant described how they tackle walking up stairs by ‘[doing] it slowly at a pace and using the stair rail… holding on to that and pulling it’ (P8) (Fig. 2b). Another participant explained that when they get very out of breath, they ‘just have to slow down’ (P3).

Participants using pacing often reflected on their experiences of hospitalisation when they ‘couldn’t sit up without being out of breath’ (P3) and used this to support their struggles of slowing down to manage the intensity of their PA. Other participants talked about how they are ‘more likely to pace’ (P10) themselves now than they used to. Some were unsure if this was ‘100%…down to the COPD’ (P10) and felt it might be ‘due to age’ (P10) or other conditions, typically thinking that ‘it’s difficult to define which [condition] is doing what’ (P13). In contrast, others indicated that they have ‘got no choice, you have to pace yourself’ (P6) due to ‘[COPD] controlling everything’ (P2) they do.

#### Subtheme 1.3: ‘Achieve it all in one go.’

**Fig. 3:**
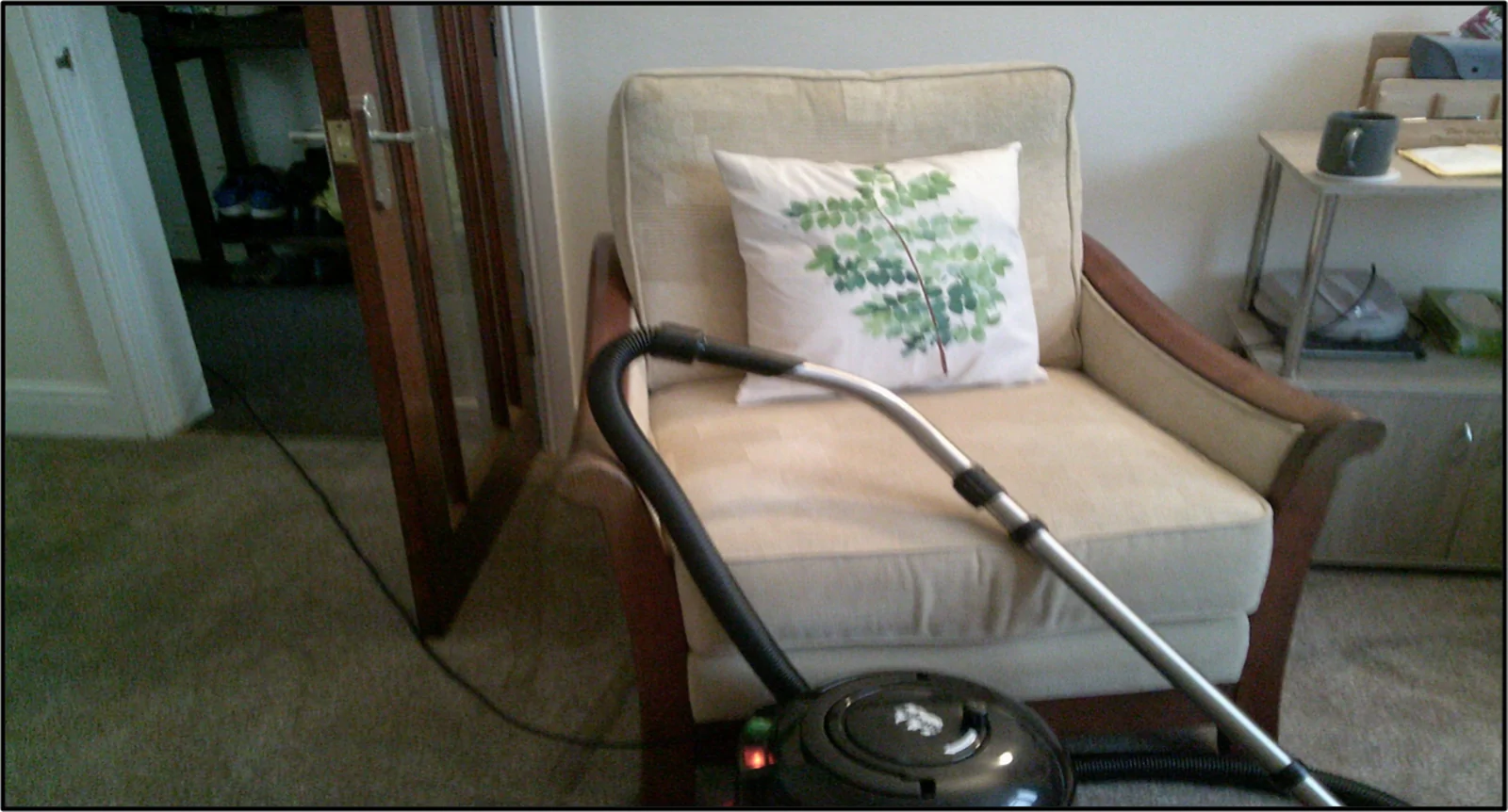
Example of doing activities all in one go, such as cleaning the whole house in one go

Several participants shared how, in some circumstances, they will deliberately do PA at a higher intensity or for longer than they found comfortable. For example, one participant explained how instead of just cleaning ‘upstairs today and then leave downstairs’ (P2) for another day, they felt they ‘can’t stop till it is done’ (P2), even if they finish and feel ‘absolutely shattered’ (P2) (Fig. 3). Another participant talked about how they ‘walked to the shop without stopping’ (P6), even when they ‘don’t feel up to going to the shop’ (P6).

Participants who attempted to complete an activity without stopping did so to gain a sense of satisfaction because ‘when it is done and you sit down and look around and… you know you have done a good job’ (P2). This was linked to an individual’s motive for doing the activity in the first place. They noted that this kind of activity created a ‘feel good’ (P2) factor for the individual. Other participants would state how there are ‘times when you…actually feel very, very healthy and fit, up to doing stuff, but then you really aren’t’ (P6). This suggested an interdependence between daily variations in health/symptoms and perceptions of their capability to do PA.

### Theme 2: Relationships between physical activity (PA) and family/friends

Theme 2 identified the links between the participants and their family/friends that can impact the PA intensity of both groups. Two subthemes were generated: (2.1) the impact of family/friends on the participants’ PA intensity and (2.2) the impact of COPD on the PA of family/friends.

#### Subtheme 2.1: Impact of family/friends on the participant’s PA intensity

**Fig. 4:**
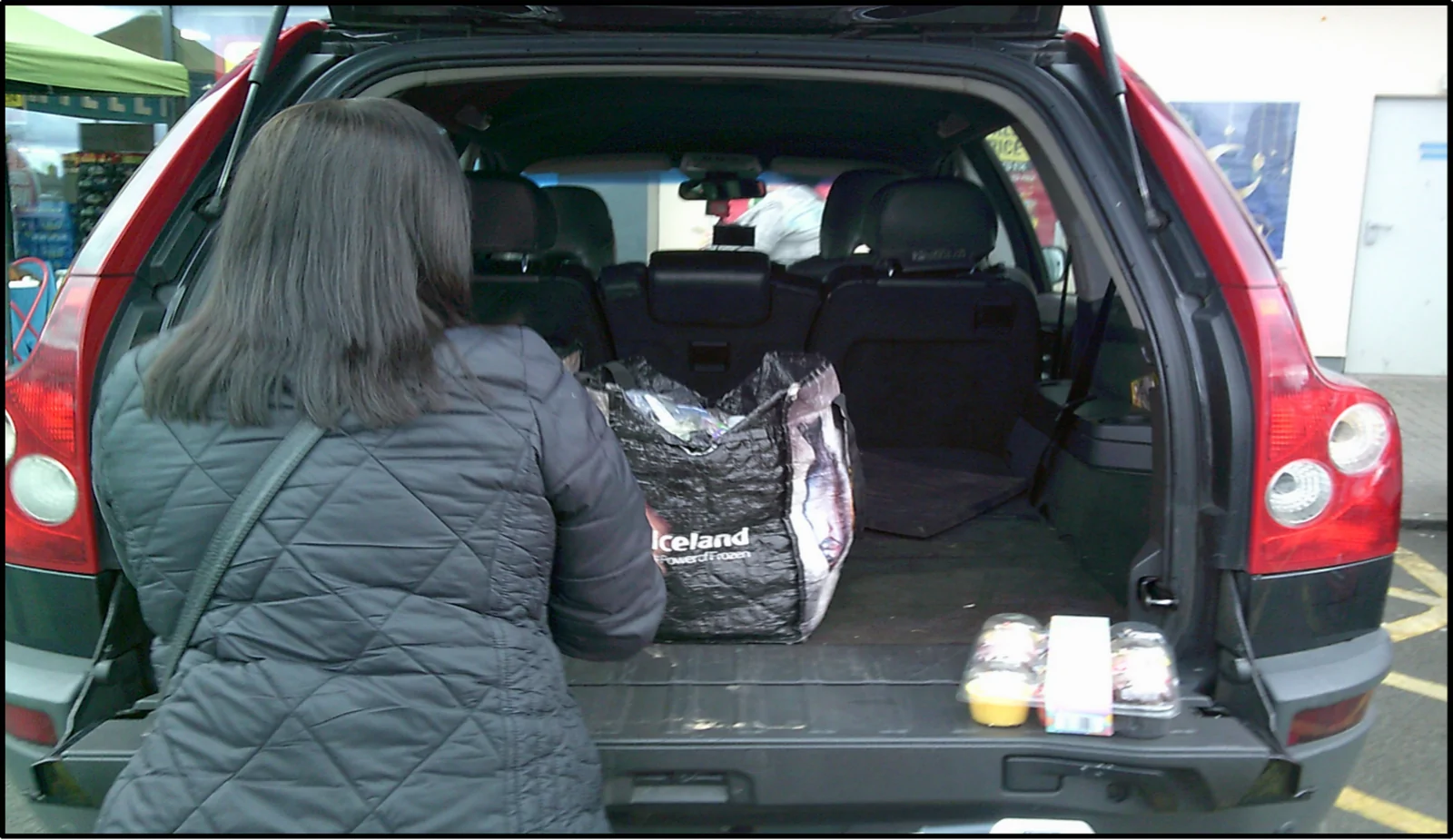
Example of how family/friends can impact a participant’s PA, such as helping with getting the shopping out of the car

Some participants spoke about the impact that family/friends have on their PA intensity. This focused primarily on ensuring they do not ‘overdo it’ (P6). One participant explained that when walking with their family, their son watches them and says ‘don’t push yourself, just sit down’ even though the participant is trying to push themselves to ‘just go a bit further’ (P2). Another participant stated that their wife is happy for them to exercise, but she worries that they are ‘doing too much’ and ‘tries to stop [them] from doing things’ (P9). In contrast, a third participant described how her children don’t understand how ‘their mother, the one who’s always done all this stuff… out on a bike… doing ten things at once… can’t do anything more or less’ (P4). A fourth participant demonstrated how their sister helps them with more physically demanding daily activities such as taking the shopping out of the car (Fig. 4).

The impact of family/friends on PA differed between respondents. In some cases, participants would reflect on how, before having COPD or other chronic conditions, their family/friends ‘would be alright’ with them doing PA but now tell them ‘not to do it’ (P6). In contrast, others acknowledged that their family/friends ‘watch out for [them]’ (P2) when doing PA so they don’t overdo it. Some participants felt ‘useless’ (P4) having to depend on family/friends to do daily activities that they were previously able to do. Some participants felt that family/friends can be ‘slightly annoying’ because they worry about the participants ‘doing too much’ and stop them from doing PA of greater intensities, even though they feel they can stop when they ‘need to stop’ (P9).

#### Subtheme 2.2: Impact of COPD on the physical activity (PA) of family/friends

**Fig. 5:**
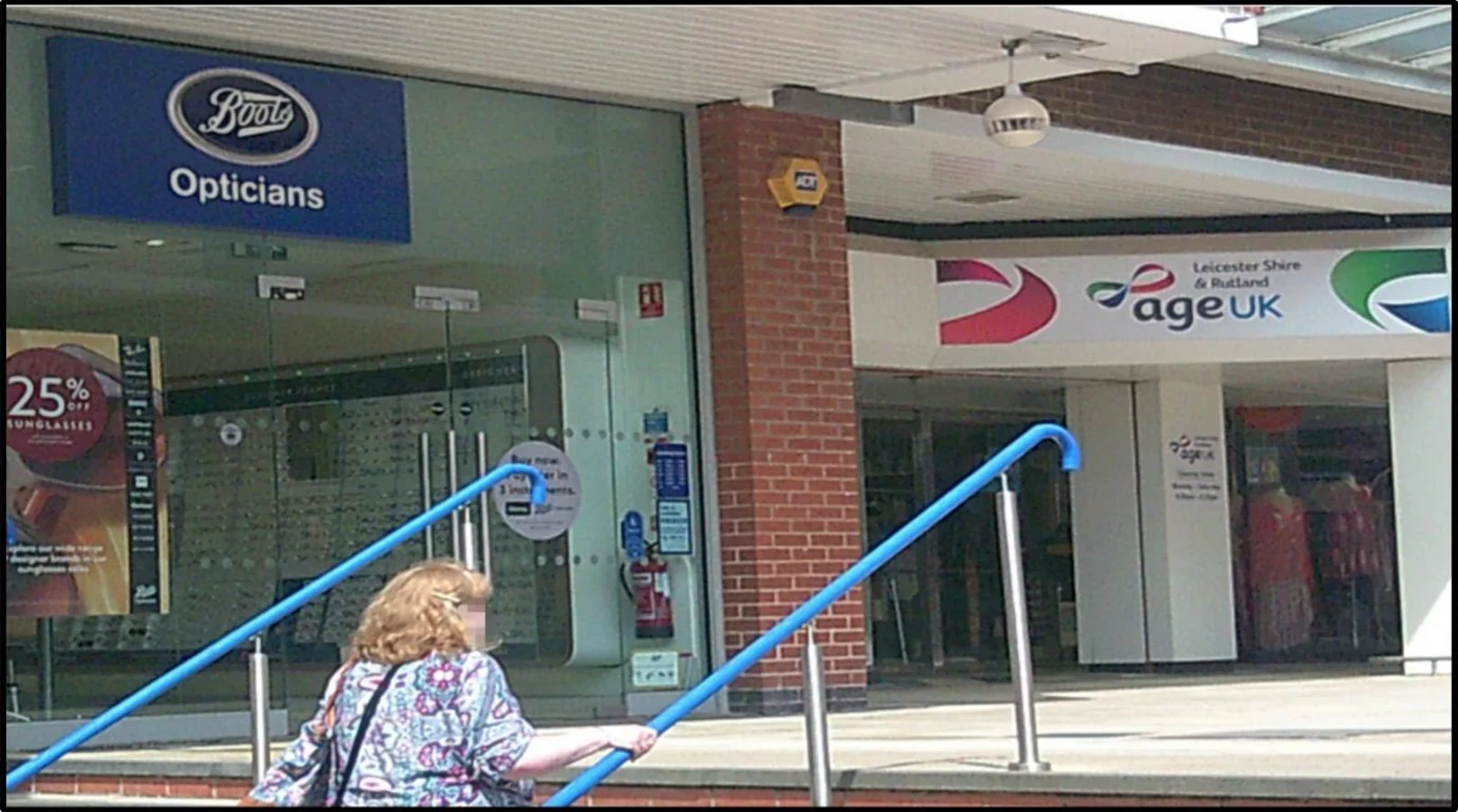
Example of the impact of chronic conditions on the PA of family/friends, such as family/friends not stopping when on a walk with a participant when they need to stop to rest and catch their breath

Some individuals reflected on how their COPD has impacted the PA of their family/friends. Effects included family/friends having to slow down, stop when doing PA, or not doing any PA at all. One participant stated, ‘I can’t do what I would like to do, and because I can’t, [husband] doesn’t either’ (P13). Another participant explained that when they walk their nephew to school and they ‘get out of breath’ (P4), they have to ask their nephew to ‘slow down’ (P4). Equally, some participants explained how family/friends do not always adjust their behaviour to accommodate their functional limitations. One participant spoke about how his girlfriend ‘doesn’t like coming out for walks’ (P12) due to the participant needing to stop and rest, resulting in him being left behind (Fig. 5).

Respondents were largely empathetic about how their condition impacted their family/friends, illustrating a degree of internalised guilt. Some participants felt that their COPD has had ‘a big impact’ (P13) on the PA of their family/friends, as they have ‘to do so much more’ (P13) to compensate for the participant’s reduced contributions to day-to-day activities. Some participants also feel that their family/friends are ‘anxious’ because of the participant’s condition, and ‘that stops them enjoying themselves when they’re around’ as they are ‘trying to protect’ the participants ‘all the time’ (P13). In contrast, others felt that family/friends have not changed their PA. For example, some participants thought it was ‘unfair to ask someone who is extremely fit… to join’ (P12) them in their PA and expect them to ‘take part in the tedious aspect of building up’ (P12) their own PA.

## Discussion

This qualitative photovoice study generated valuable insights into the real-life context and the different approaches individuals with COPD use to manage the intensity of their daily PA, building on previous PA photovoice research [21, 22]. These included breaking up activity with rest to prevent the intensity of PA going too high, pacing themselves by downgrading the intensity of an activity, or trying to do an activity all in one go, even at high intensities, to get it over and done with. This approach often resulted in the participants wearing themselves out and then having no energy to do any other activity. This study also highlighted the impact of family/friends on the PA of an individual with COPD by ensuring they do not overextend themselves through daily activities. Equally, an individual’s condition can impact the PA of family/friends, stopping them from taking part in activities their friends/relatives with COPD find too intense. Despite research to date focusing on quantitative examinations [7, 32–34], the data presented here provide a greater understanding of relative intensity in the ‘real world’, as it is an intrinsically qualitative concept.

In combination with previous research, our findings can inform future interventions and be integrated into existing clinical services, such as PR. For example, integration might occur in the educational components of classes or in discussions between healthcare professionals and patients during their initial assessments or rehabilitation programmes, such as through motivational interviewing techniques [35, 36]. These techniques could assist healthcare professionals in identifying patients’ perceptions and strategies to manage the intensity of their PA, and in evaluating whether these strategies are safe and appropriate by comparing them with current guidelines. Additionally, these techniques can help patients recognise and discuss potentially inappropriate strategies used to manage their daily activities, such as attempting to do an activity ‘all in one go’. Instead, patients could be encouraged to use alternative methods recommended in PR, such as pacing [37], which will help them maintain the meaning behind their daily tasks.

### Theme 1: Approaches to PA Intensity

Current treatment for individuals with COPD primarily focuses on symptom management, particularly addressing dyspnoea during daily activities [9]. Guidelines for PR in individuals with COPD emphasise the importance of educating individuals on energy conservation strategies (prioritising activities, planning ahead, and pacing oneself) during daily activities, which can also support symptom management [37]. These behaviours align with two of the approaches identified in this study: ‘Break it down’ and ‘Pacing myself’. However, the final approach of ‘Achieve it all in one go’ appears to be the opposite of the other approaches. This approach may lead some individuals with COPD to experience a more extended recovery period before resuming other daily activities. The use of this approach often depended on the activity the participants were taking part in and could also be associated with a lack of acceptance of the illness. For example, individuals with COPD often struggle with accepting their disease, hindering their willingness to adjust their PA and continuing with their normal levels of PA, exacerbating their condition further [38]. Acceptance is essential for adapting to life with COPD, as it fosters a positive attitude toward the condition and the therapeutic process while influencing overall life satisfaction [39].

The motive for engaging in PA was identified as a crucial factor influencing the self-management of PA intensity. This finding is consistent with prior research highlighting self-motivation as a key element that facilitates participation in PA [40]. Motives for engaging in PA may include the desire to play with grandchildren, goals related to weight maintenance, and preparations for upcoming events [40]. Additionally, fluctuations in daily symptoms were identified as a factor that affects how an individual feels daily, corroborating research by Miravitlles et al. [10] and Johansson et al. [41]. This variability could subsequently influence the choice of the approach used to manage the intensity of daily activities.

In this study, participants frequently engaged in social comparisons concerning their ability to participate in PA. Several participants emphasised the impact of age on their current capacity for PA. Quantitative research indicates that the percentage of older adults who meet national guidelines for moderate to high-intensity PA on an absolute intensity scale declines with age [42]. However, when activity is expressed relative to their physical capacity, the proportion of older adults meeting these guidelines increases with age [42]. Although the influence of age was highlighted by some participants in the present study, the majority of individuals contrasted their current self, living with COPD, against their previous, healthier selves (free from COPD) rather than focusing on age-related changes. This shows that changes in perceived intensity extend beyond ageing, underscoring the need for earlier intervention for those living with COPD.

When age was discussed, it was typically to compare themselves with peers of a similar age who are free from COPD, establishing a benchmark for evaluating their own performance that poorly reflects the challenges of this particular condition. This concept is evident in the way PA intensity has been typically measured in quantitative research [6, 9–11], primarily through the use of accelerometers and intensity cutpoints derived from data on predominantly young-to-middle-aged adults, who possess a greater physical capacity than those with most chronic conditions. This means that existing cutpoints are largely inappropriate for individuals with COPD [43]. Our study highlights the same discrepancy and demonstrates how social comparisons can profoundly impact one’s self-perception and understanding of their condition [44, 45].

### Theme 2: Relationships between their PA and family/friends

The findings from this study add to prior research indicating that families of patients with COPD may exhibit protective behaviours, either actively or passively influencing the individual’s decision to disengage from PA [46–48]. These behaviours are likely to restrict higher-intensity activities, which are associated with greater health benefits [4]. Conversely, some family/friends may struggle to comprehend why individuals with COPD cannot maintain their former levels of activity, as they tend to perceive them through the lens of their past, healthier selves. Previous studies have highlighted the significant role that family members play in supporting people with COPD as they adapt their lives and engage in self-management behaviours [44, 45, 49, 50]. However, as demonstrated in this study, dependence on family members can lead to feelings of guilt and frustration associated with this reliance, as well as shifts in family dynamics.

Several participants in this study mentioned how their COPD directly impacts the PA of other people, with family/friends having to slow down, interrupt, or refrain from engaging in PA. This finding aligns with previous qualitative research [51, 52], which has demonstrated that the daily activities of family members of individuals with COPD can be affected, with some family members having to limit their own activities. This was due to the individuals with COPD having a diminished functional status, where activities of daily living were affected and required assistance from their family members [51–53].

### Strengths and Limitations

This study has several strengths. The use of photovoice enhanced qualitative discussion, capturing rich and meaningful data on participants’ experiences of PA intensity and allowing for a comprehensive consideration of reflexivity in research. Additionally, participants directed the flow of the interview conversations by choosing their photographs rather than an interview schedule pre-defined by the research team, making the use of photovoice more empowering compared to traditional interview methods. This approach may have also helped participants feel less intimidated and more willing to share their thoughts, leading to more authentic and accurate data collection. The use of photovoice also benefited those who found it difficult to express complex issues, and thus it provided a more inclusive study design and less hierarchical research process. Moreover, the study sample was a comorbid group with symptom burden that impacts their daily life, meaning the results from this study likely apply to other populations with similar comorbidities. However, the use of photovoice could introduce sampling bias, as it tends to favour individuals who possess the ability or willingness to use a camera. Furthermore, photographs may not adequately represent the full complexity of the observed situation and could exhibit limitations in their generalisability to diverse populations or contexts. Our study limitations included only having one participant with the highest level of breathlessness burden (MRC 5). For these individuals, the approaches to PA intensity may look different, given the potential for limitations in personal care (dressing/undressing self) or being unable to leave their home. The majority of participants in this study were White British. Whilst this demographic reflects the typical population in UK-based COPD research [54], future work is needed to explore perceptions of PA intensity across more diverse cultures and ethnicities.

### Implications for research and clinical practice

The current research offers a comprehensive picture of the different approaches used by individuals with COPD when tackling higher-intensity PA and explores the relationship between family/friends and PA. Using photovoice in this study has provided evidence of what is important to these individuals. In contrast, textual analyses of data are driven in part by what the researcher deems important [16]. These insights should be used to inform interventions to support people living with COPD to participate in higher-intensity PA. The findings of this study emphasise that there is no “one size fits all” approach to PA intensity, underlining the need for an individualised and targeted strategy for PA prescription, monitoring and evaluation, as well as family/friends being involved in the care process of individuals with COPD, particularly concerning their PA. Such insights can facilitate discussions about PA between healthcare professionals and patients about how to modify their current strategies to tackle their PA, enabling engagement in higher (relative) intensity activities, and educating individuals and their support networks on engaging in higher-intensity PA.

## Conclusion

Drawing on in-depth qualitative interviews augmented with photovoice techniques, to our knowledge, this study is the first to examine PA intensity in COPD using photovoice. This study has identified different approaches employed by individuals with COPD to manage the intensity of PA. These approaches include breaking down activity, pacing themselves, or trying to do the activity all in one go. Family/friends can often impact the PA of an individual with COPD by ensuring they do not do too much or by helping them with daily activities. Equally, an individual’s condition can impact the PA of family/friends, stopping family/friends from taking part in activities that the individual with COPD finds relatively intense. The findings from this study could be drawn upon to develop interventions, which may include facilitating discussions about PA between healthcare professionals and patients, promoting engagement in relatively higher-intensity activities and education strategies on how to boost participation in higher-intensity PA.

## Supplementary Information

Supplementary Material 1.

## Data Availability

Summarised data analysed for this study are included within the text and table of this article.
