# Supplementary material for: Intensity is in the eye of the beholder: a qualitative photovoice study of perceptions of physical activity intensity for people with chronic obstructive pulmonary disease
Source: BMC Pulm Med. 2026 Jul 21;26:479. doi: 10.1186/s12890-026-04520-8 (PMC13673597; doi:10.1186/s12890-026-04520-8)
Supplement: Supplementary file 1 [file 12890_2026_4520_MOESM1_ESM.docx]

**Supplementary material for manuscript Lloyd-Evans et al. “Intensity is in the eye of the beholder: A qualitative photovoice study of perceptions of physical activity intensity for people with chronic respiratory disease”**

**Table S1 -** Consolidated criteria for reporting qualitative studies (COREQ): 32-item checklist

**Table S2 –** Research Interview Schedule

**Table S1 -** Consolidated criteria for reporting qualitative studies (COREQ): 32-item checklist

Developed from: Tong A, Sainsbury P, Craig J. Consolidated criteria for reporting qualitative research (COREQ): a 32-item checklist for interviews and focus groups. *International Journal for Quality in Health Care*. 2007. Volume 19, Number 6: pp. 349 – 357

| **No. Item** | **Guide questions/description** | **Reported on Page #** |
| --- | --- | --- |
| **Domain 1: Research team and reﬂexivity** | | |
| *Personal Characteristics* | | |
| 1. Interviewer/facilitator | Which author/s conducted the interview or focus group? | 6 |
| 2. Credentials | What were the researcher’s credentials? E.g., PhD, MD | 6 & 7 |
| 3. Occupation | What was their occupation at the time of the study? | 5 |
| 4. Gender | Was the researcher male or female? | 6 |
| 5. Experience and training | What experience or training did the researcher have? | 6 |
| *Relationship with participants* | | |
| 6. Relationship established | Was a relationship established prior to study commencement? | 5-6 |
| 7. Participant knowledge of the interviewer | What did the participants know about the researcher? e.g., personal goals, reasons for doing the research | 5-6 |
| 8. Interviewer characteristics | What characteristics were reported about the interviewer/facilitator? e.g., Bias, assumptions, reasons and interests in the research topic | 6, 8 & 9 |

| **Domain 2: study design** | | |
| --- | --- | --- |
| *Theoretical framework* | | |
| 9. Methodological orientation and Theory | What methodological orientation was stated to underpin the study? e.g., grounded theory, discourse analysis, ethnography, phenomenology, content analysis | 5 |
| *Participant selection* | | |
| 10. Sampling | How were participants selected? e.g., purposive, convenience, consecutive, snowball | 5-6 |
| 11. Method of approach | How were participants approached? e.g., face-to-face, telephone, mail, email | 5 |
| 12. Sample size | How many participants were in the study? | 9 |
| 13. Non-participation | How many people refused to participate or dropped out? Reasons? | 9 |
| *Setting* | | |
| 14. Setting of data collection | Where was the data collected? e.g., home, clinic, workplace | 6-7 |
| 15. Presence of non-participants | Was anyone else present besides the participants and researchers? | N/A |
| 16. Description of sample | What are the important characteristics of the sample? e.g., demographic data, date | 9 & Table 1 |
| *Data collection* | | |
| 17. Interview guide | Were questions, prompts, and guides provided by the authors? Was it pilot-tested? | 6 & Supplementary material (Table 2) |
| 18. Repeat interviews | Were repeat interviews carried out? If yes, how many? | N/A |
| 19. Audio/visual recording | Did the research use audio or visual recording to collect the data? | 6-7 |
| 20. Field notes | Were ﬁeld notes made during and/or after the interview or focus group? | 7 |
| 21. Duration | What was the duration of the interviews or focus group? | 9 |
| 22. Data saturation | Was data saturation discussed? | 8 |
| 23. Transcripts returned | Were transcripts returned to participants for comment and/or correction? | 7 |
| **Domain 3: analysis and ﬁndings** | | |
| *Data analysis* | | |
| 24. Number of data coders | How many data coders coded the data? | 7 |
| 25. Description of the coding tree | Did the authors provide a description of the coding tree? | N/A |
| 26. Derivation of themes | Were themes identiﬁed in advance or derived from the data? | 7 |
| 27. Software | What software, if applicable, was used to manage the data? | N/A |
| 28. Participant checking | Did participants provide feedback on the ﬁndings? | N/A |
| *Reporting* | | |
| 29. Quotations presented | Were participant quotations presented to illustrate the themes/ﬁndings? Was each quotation identiﬁed? e.g., participant number | 10-18 |
| 30. Data and ﬁndings consistent | Was there consistency between the data presented and the ﬁndings? | 10-18 |
| 31. Clarity of major themes | Were major themes clearly presented in the ﬁndings? | 10-18 |
| 32. Clarity of minor themes | Is there a description of diverse cases or discussion of minor themes? | 10-18 |

**Table S2 – Research Interview Schedule**

| Focus | Examples of questions and probes |
| --- | --- |
| Health condition - COPD | Ice Breaker and Re-Consent – 3-5 minutes   - Can you tell me a little about yourself? Education, work family, hobbies.   About their COPD   - Tell me a little about your COPD and how you cope with it (daily basis). - Can you describe what a good day looks like and what a bad day looks like? - What makes the COPD worse – Weather, pollen, doing certain activities? - How has COPD impacted other aspects of your life? - Currently, what are your main problems? Concrete examples of how it effects daily living – what can’t do but feel should be able to do. (First probe different areas – work, household duties, leisure time, strain on relations with others. But *ultimately the patient may say they do not have any problems, and this is fine.)* - How would you describe your approach to having COPD? Fighting it head on? Getting by? Muddling along? Or too busy with other things to really think about COPD? How would you put it in your own words? |
| Perceptions of physical activity and relative intensity | Treatment of COPD   - How are you being treated for COPD – Medication/Pulmonary rehabilitation? - Have you been advised to be more physically active? - *If so, who by (doctor, family, friend?) and what did they advise (just do what you can? More is better? Etc.)* - What were your initial thoughts about being more physically active? What did you think you should do? What kinds of things were suggested you do? What do you think you should avoid? Have you been told to avoid anything in particular? - What is your previous history of doing physical activity before you were diagnosed? Did you play sport as a child? Or at all through being an adult. Have you had jobs that were particularly active? - Currently, do you take part in any regular physical activity and if so, can you give me any examples of what physical activity? What would you say was the most physically active thing that you regularly do? - What do you feel before, during and after physical activity (physical, emotional, psychological symptoms)? - How would do describe your approach in doing exercise, housework, gardening etc.? Do you throw yourself into it? Push but with caution? Pace yourself? Best avoided? - Can you be too active? How would that impact on your COPD/other conditions? - How has the intensity of your physical activity/daily activities changed since you were diagnosed with COPD? How does this differ to before you were diagnosed? - When you are exercising, what do you take as good signs? Sweating, breathless, tired, Having fun? - What positive experiences have you had from doing physical activity that may have been more moderate/ vigorous physical activity? - What negative experiences have you had from doing physical activity that may have been more moderate/ vigorous physical activity? - Are you satisfied with your activity levels, or would you like to do more or less? If you’re not, why not? What could be different? What would help you make it different? What are the difficulties you face in being active? - How has your COPD or other comorbidities changed your perceptions of physical activity and doing specific activities? Something you are or should be doing more or less regularly? More or less intensively?   Family and friends   - What do those around you think about you doing exercise? - Are others in your family/friends very active? - Do they see physical activity as a waste of time for someone with COPD to exercise? - How do they encourage/discourage you from doing physical activity? - Do you do activities with others or along. And if with others who and why? |
| Photovoice | Referring to images taken:   - When was this taken? - What were you doing at the time? And who with? - Who and/or what is in the photo? - What did you want to convey with these images? - What made you want to take those photographs? - How did you feel at the time when taking the photos? - Why is it important for others to know about this? - What does this say that other people might not understand about your life with COPD? - Who would you want to share this photo with, and how do you think they would respond? How would you want them to respond? - Would they see it in the same way as you or differently? Is that important, and if so, why? - What challenges have you experienced over the last eight days? |
| Reflections and final questions | Reflections   - What are your reflections on being interviewed? - Was that what you were expecting? - How do you think the rest of the research will go? - Can you help me to make my study better? - Following this interview, do you feel you have been encouraged/discouraged to take part in other phases? |
